# Association Between Preoperative Psychological Distress and Successful Weight Loss After Bariatric Surgery: A Retrospective Study

**DOI:** 10.3390/jcm14207333

**Published:** 2025-10-17

**Authors:** Warut Aunjitsakul, Kamthorn Yolsuriyanwong, Siripong Cheewatanakornkul, Darawan Promchan, Chaitong Churuangsuk

**Affiliations:** 1 Department of Psychiatry, Faculty of Medicine, Prince of Songkla University, Songkhla 90110, Thailand; warut.a@psu.ac.th; 2 Songklanagarind Excellence Center for Obesity and Metabolic Surgery, Department of Surgery, Faculty of Medicine, Prince of Songkla University, Songkhla 90110, Thailand; kamthorn.y@psu.ac.th (K.Y.); siripong.c@psu.ac.th (S.C.); 3 Nursing Service Division, Songklanagarind Hospital, Prince of Songkla University, Songkhla 90110, Thailand; pdarawann@hotmail.com; 4 Clinical Nutrition and Obesity Medicine Unit, Department of Internal Medicine, Faculty of Medicine, Prince of Songkla University, Songkhla 90110, Thailand

**Keywords:** mental health, psychological distress, General Health Questionnaire, preoperative assessment, bariatric surgery, weight reduction

## Abstract

**Objectives**: The relationship between preoperative psychological distress and weight loss following bariatric surgery remains limited in Asian populations. This study aimed to investigate whether preoperative psychological distress, as a general screening measure, predicted weight loss following bariatric surgery in a Thai population. **Methods**: We conducted a retrospective cohort study of 464 patients who underwent bariatric surgery at a university hospital between 2020 and 2023. Preoperative psychological distress was assessed using the General Health Questionnaire-28 (GHQ-28), with a score of ≥6 indicating high psychological distress. The primary outcome was successful weight loss (SWL), defined as achieving >50% excess weight loss at 6 and 12 months postoperatively. We used multivariable logistic regression models, adjusted for age, sex, surgery type, obesity-related comorbidities, and baseline body weight, to analyze the association between psychological distress and SWL outcomes. **Results**: Patients with high psychological distress (*n* = 270) demonstrated significantly higher rates of SWL compared to those with low distress (*n* = 194) at both 6 months (59.7% vs. 43.5%, *p* = 0.003) and 12 months (83.6% vs. 74.6%, *p* = 0.068). In adjusted regression analyses, patients with high distress had approximately twice the odds of achieving SWL at 6 months (adj. OR 1.99, 95% CI: 1.25–3.17, *p* = 0.004), with this association persisting at 12 months (adj. OR 1.86, 95% CI: 1.02–3.39, *p* = 0.044). Subgroup analyses revealed consistent associations across both sexes, with no significant interaction effects. **Conclusions**: Contrary to traditional assumptions, higher preoperative psychological distress was associated with greater odds of achieving successful weight loss after bariatric surgery. This suggests that psychological distress may not be a barrier to successful outcomes but could, when supported appropriately, be a predictor for significant weight loss. These findings highlight the value of psychological assessment in optimizing, rather than restricting, bariatric surgery candidates.

## 1. Introduction

Obesity has emerged as a global health crisis, affecting over 890 million adults worldwide and imposing substantial economic burdens on healthcare systems [1,2]. This complex medical condition, characterized by excessive adipose tissue accumulation, results from intricate interactions between genetic predispositions, environmental factors, and individual behaviors [2,3]. Its effects go beyond physical health, leading to severe health problems, including type 2 diabetes, cardiovascular diseases, and certain cancers [4,5]. Obesity also exerts a profound impact on psychological well-being, contributing to heightened risks of depression and diminished self-image [6,7]. The financial burden on healthcare systems, including both medical costs and lost productivity, emphasizes the need for comprehensive solutions, including lifestyle changes, better access to healthy food, increased physical activity, and supportive health policies [1,2,8].

Management of obesity involves a multidisciplinary approach aimed at addressing its complex etiology and associated health risks. Behavioral interventions, such as dietary modifications, increased physical activity, and behavioral therapy, form the cornerstone of non-surgical management strategies [8,9]. These approaches emphasize sustainable lifestyle changes and are often complemented by pharmacotherapy in cases where lifestyle modifications alone are insufficient [9]. Bariatric surgery, a highly effective intervention for severe obesity, is considered when other treatments have failed to achieve desired weight loss or when obesity-related comorbidities are particularly severe [10,11]. Common procedures such as gastric bypass, sleeve gastrectomy, and adjustable gastric banding aim to reduce stomach capacity or alter the gastrointestinal tract, resulting in significant and sustained weight loss as well as improvements in obesity-related comorbidities [10]. However, optimal outcomes require careful patient selection, comprehensive preoperative assessment, and long-term postoperative management to mitigate potential risks [10,11].

Candidates for bariatric surgery often undergo comprehensive psychological evaluation to assess their readiness for the procedure and to identify potential psychological factors that may impact their postoperative outcomes [6]. Issues such as body image dissatisfaction, emotional eating patterns, depression, anxiety, and substance use are systematically evaluated due to their potential influence on adherence to postoperative dietary and lifestyle recommendations, as well as overall treatment success [6,7]. Studies indicate that 19–66% of bariatric surgery candidates present with psychological conditions that may influence surgical outcomes [6,12].

The postoperative outcomes of bariatric surgery vary depending on several factors, including the type of procedure performed, the patient’s preoperative health status, adherence to postoperative guidelines, and the presence of comorbidities [11,13]. Research has demonstrated that psychological assessment is crucial for surgery success and patient’s well-being [14]. Weight loss outcomes typically show a gradual decline in excess body weight over the first 12 to 18 months following surgery, with most patients achieving their maximum weight loss within the first two years [10].

While psychological assessment is widely recommended before bariatric surgery [15], the relationship between preoperative psychological distress and postoperative outcomes remains inconsistent in the literature [16,17]. A systematic review by Dawes et al. (2016), Al-Kadi et al. (2024) and Jacobs et al. (2024) found that preoperative psychological conditions had varying associations with weight loss outcomes, with some studies showing negative impacts, while others showed no significant effects [12,16,18]. Additionally, most previous research has focused on specific psychiatric diagnoses rather than general psychological distress, which may be more prevalent in bariatric candidates. Therefore, this study aimed to investigate the association between preoperative psychological distress, measured using a validated general screening tool, and weight loss outcomes in an Asian population. We hypothesized that higher levels of psychological distress would be associated with worse post-bariatric surgery outcomes.

## 2. Materials and Methods

### 2.1. Study Design and Population

A retrospective cohort study was conducted by reviewing the electronic medical records of all patients who underwent bariatric surgery between January 2020 and December 2023 at Songklanagarind Hospital. This university teaching hospital is affiliated with the Faculty of Medicine, Prince of Songkla University, Thailand. The study protocol received ethical approval from the Institutional Human Research Ethics Committee of the same institution. All patients were managed under a consistent multidisciplinary protocol as part of our center’s comprehensive care. This team included a bariatric surgeon, a dietitian, a psychiatrist, an otolaryngologist, and an internal medicine specialist (specializing in clinical nutrition). The standardized care from this team ensured that all patients received a comprehensive preoperative evaluation and were managed similarly throughout the study period.

Participant inclusion criteria were: (1) adults aged 18 years or older; (2) having undergone primary bariatric surgery during the study period; (3) completion of preoperative psychological assessment; and (4) availability of weight measurements at baseline and at least one follow-up visit (6 or 12 months postoperatively). Exclusion criteria included: (1) previous bariatric surgery; (2) preexisting diagnosis of severe psychiatric disorders (e.g., active psychosis, severe depression with suicidal ideation, or substance use disorders); (3) incomplete psychological assessment data; and (4) loss to follow-up before the first postoperative visit. Severe psychiatric disorders were excluded because their active symptoms, such as poor self-control or inability to perform wound care, could significantly impact both pre-operative and post-operative care, potentially leading to fatal outcomes. This exclusion was a necessary step to ensure the safety of the participants.

### 2.2. Demographic Data

The study collected brief demographic data including sex, age, preoperative body weight, height, obesity-related comorbidities, and types of bariatric surgery. Body mass index (BMI) was also included and calculated by dividing weight in kilograms by height in meters squared. Obesity-related comorbidities were identified through medical record review based on documented diagnoses, following the standardized framework for metabolic and bariatric surgery outcomes reporting [19]. All diagnoses of comorbidities were recorded in the electronic medical records prior to surgery. Comorbidities included: type 2 diabetes mellitus (defined by HbA1c ≥ 6.5% or use of antidiabetic medications), hypertension (defined by blood pressure > 140/90 mmHg or use of antihypertensive medications), dyslipidemia (defined by high LDL cholesterol, high triglycerides or use of lipid-lowering medications), obstructive sleep apnea (diagnosed by polysomnography), gastroesophageal reflux disease (diagnosed clinically or by endoscopy), metabolic-associated steatotic liver disease (diagnosed by ultrasonography), and impaired fasting glucose (fasting glucose 100–125 mg/dL).

### 2.3. Exposure of Interest

The study exposure was psychological distress status, assessed at baseline (preoperatively) using the General Health Questionnaire—28 (GHQ-28), a validated screening tool for psychological distress [20]. All candidates for bariatric surgery underwent standardized psychological evaluation as part of our center’s multidisciplinary assessment protocol and completed the GHQ-28, with possible total scores ranging from 0 to 28. We used a GHQ-28 threshold of ≥6 to define high psychological distress, consistent with the conventional cutoff validated in general populations. The GHQ-28 demonstrated excellent psychometric properties with a Cronbach’s alpha of 0.91, indicating high reliability [21].

### 2.4. Outcomes of Interest

#### 2.4.1. Primary Outcome

The primary outcome was successful weight loss (SWL) at 6 and 12 months post-bariatric surgery, defined as losing more than half of the excess weight. SWL was chosen as the primary outcome because it represents a clinically meaningful threshold widely used in bariatric surgery literature to assess treatment efficacy. The excess body weight was calculated by subtracting individuals’ ideal body weight from their baseline body weight.

#### 2.4.2. Secondary Outcome

Percentage total weight loss (%TWL) at 6 and 12 months was examined as a secondary outcome to provide additional context for the magnitude of weight reduction. %TWL provides a standardized measure to compare weight loss outcomes across patients with different initial body weights and has been shown to have stronger associations with improvements in obesity-related comorbidities.

### 2.5. Covariates

Given the objective of the study to evaluate the association between psychological distress and weight loss outcome, we pre-specified the following covariates: age, sex, type of surgery, preoperative body weight, and number of obesity-related comorbidities. These covariates were selected based on their established associations with post-bariatric surgery weight loss outcomes in previous literature. Advanced age has been shown to affect weight loss trajectory, while sex differences influence both baseline metabolism and post-surgical outcomes. The type of surgical procedure directly impacts the degree of weight loss through different anatomical and physiological mechanisms. Higher preoperative body weight is known to affect the absolute amount of weight loss possible, and the number of obesity-related comorbidities can influence both surgical approach and post-operative recovery patterns.

### 2.6. Statistical Analysis

Descriptive statistics were presented as frequency (percentage) for categorical variables and mean (SD) or median (IQR) for continuous variables. Comparisons between high- and low-psychological-distress groups (GHQ ≥ 6 vs. <6) were performed using chi-square test for categorical variables, and *t*-test or Mann–Whitney U test for continuous variables.

For primary analysis, the association between psychological distress status and the primary outcome (SWL) was assessed using multivariable logistic regression, adjusted for pre-specified covariates: age, sex, type of surgery, preoperative body weight, and number of obesity-related comorbidities.

While outcomes were assessed at two time points (6 and 12 months), these represent a longitudinal trajectory of the same clinical endpoint rather than independent hypotheses. The 6-month assessment captures early postoperative weight loss, while the 12-month assessment reflects more sustained outcomes. Therefore, no adjustment for multiple comparisons was applied to the primary analyses. We reported both time points to demonstrate the temporal pattern of the association.

Given the observed sex imbalance in our cohort (75.6% female), we conducted post hoc subgroup analysis stratified by sex. We also tested for statistical interaction between sex and GHQ status by including an interaction term (sex × GHQ status) in the regression models to assess whether the association between psychological distress and weight loss outcomes differed by sex.

We also conducted post hoc power analysis, and as this was a retrospective cohort study, sample size was determined by the number of eligible patients who underwent bariatric surgery during the study period (January 2020 to December 2023). Post hoc power analyses were conducted using the WebPower package in R version 4.4.1 to assess whether our sample size was adequate for detecting clinically meaningful associations. Power calculations for the primary outcome (SWL) were based on logistic regression analyses with the following parameters: observed odds ratios, baseline success proportions in the low-distress group, total sample sizes at each time point, and a two-tailed significance level of α = 0.05. At 6 months (*n* = 367), with an observed odds ratio of 1.99 and baseline success proportion of 43.5% in the low-distress group, achieved power was 90.1%. At 12 months (*n* = 315), with an observed odds ratio of 1.86 and baseline success proportion of 74.6%, achieved power was 58.2%.

Minimum detectable effect size analyses indicated that our study at 6 months could detect a difference of 15 percentage points in success rates with 80% power, and 17 percentage points with 90% power. At 12 months, the study could detect differences of 12 and 14 percentage points for 80% and 90% power, respectively. The observed difference at 6 months (16.2 percentage points) exceeded the minimum detectable effect for 80% power, while the observed difference at 12 months (9.0 percentage points) was smaller than the minimum detectable effect, consistent with the lower achieved power at this time point.

All statistical analyses were performed using R version 4.4.1 with *p* < 0.05 considered statistically significant.

## 3. Results

### 3.1. Study Population

A total of 464 patients underwent bariatric surgery between January 2020 and December 2023. Follow-up data, collected from this cohort, was completed for 367 patients at 6 months and 315 patients at 12 months. The majority of patients were female (75.6%, *n* = 351), with a median age of 34 years (interquartile range [IQR] 28–42; Table 1). Regarding surgical procedures, sleeve gastrectomy was the most performed procedure (73.7%, *n* = 342), followed by gastric bypass (14.2%, *n* = 66) and single anastomosis duodeno-ileal bypass (SADI-S, 12.1%, *n* = 56). Procedures were performed using laparoscopic or robotic approaches, with some cases including concurrent operations such as hiatal hernia repair. At baseline, the median preoperative body weight was 117 kg (IQR 103.2–135.8) with a corresponding median BMI of 43.9 kg/m^2^ (IQR 39.4–50.0; Table 2).

The majority of patients (91.4%) presented with at least one obesity-related comorbidity, with approximately one-fifth (19.2%) having three concurrent comorbidities. The most prevalent comorbidities included dyslipidemia (36.6%), hypertension (33.0%), and metabolic associated steatotic liver disease (32.1%).

### 3.2. Baseline Psychological Assessment and Group Characteristics

Analysis of preoperative psychological status revealed a median GHQ score of 7 (IQR 3–13) across the entire cohort (Table 1). Using the conventional threshold of 6, we classified 270 patients (58.2%) as having high psychological distress (GHQ ≥ 6) and 194 patients (41.8%) as having low psychological distress (GHQ < 6).

When comparing baseline characteristics between groups, both groups showed similar distributions in terms of age (median 33 vs. 34 years, *p* = 0.798), preoperative body weight (median 115 vs. 118.9 kg, *p* = 0.056), and BMI (median 43.3 vs. 44.6 kg/m^2^, *p* = 0.124). Patients with high psychological distress were more likely to be female (79.6% vs. 70.1%, *p* = 0.025). The distribution of surgical procedures (*p* = 0.154) and the number of obesity-related comorbidities (*p* = 0.641) were also comparable between groups.

### 3.3. Successful Weight Loss (Primary Outcome)

At the 6-month follow-up (*n* = 367), 193 patients (52.6%) achieved SWL, defined as losing more than 50% of excess body weight. By 12 months post-surgery (*n* = 315), the proportion of patients achieving SWL substantially increased to 79.7% (*n* = 251), reflecting the continued weight loss trajectory in the first postoperative year (Table 2).

When stratified by psychological distress status, patients with high distress demonstrated significantly higher rates of SWL compared to those with low distress. At 6 months, 59.7% of high-distress patients achieved SWL compared to 43.5% of low-distress patients (*p* = 0.003). At 12 months, the difference remained apparent, with 83.6% of high-distress patients achieving SWL compared to 74.6% of low-distress patients (*p* = 0.068).

### 3.4. Percentage Total Weight Loss (Secondary Outcome)

Mean %TWL at 6 months was 25.8% (SD 6.1), which increased to 31.6% (SD 7.3) at 12 months. Patients with high psychological distress achieved similar %TWL compared to those with low distress at both 6 months (26.4% vs. 25.1%, *p* = 0.054) and 12 months (32.3% vs. 30.7%, *p* = 0.055), with differences that were neither statistically nor clinically significant. For absolute weight loss, the differences between groups were not statistically significant at either time point (6 months: 29.3 kg vs. 29.5 kg, *p* = 0.912; 12 months: 36.2 kg vs. 37.2 kg, *p* = 0.627).

### 3.5. Association Between Psychological Distress and Successful Weight Loss

**Figure 1:**
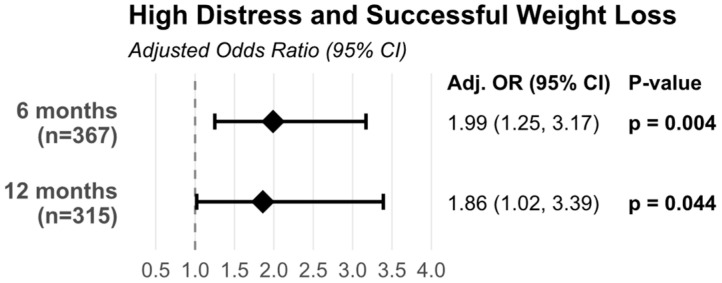
Adjusted odds ratio of high distress and successful weight loss. Regression models were adjusted for age, sex, surgery type, number of obesity-related comorbidities, and baseline body weight.

In multivariable logistic regression analyses adjusted for age, sex, surgery type, preoperative body weight, and number of obesity-related comorbidities, patients with high psychological distress (GHQ ≥ 6) had significantly greater odds of achieving successful weight loss compared to those with low distress (Figure 1 and Table A1).

At 6 months post-surgery, the adjusted odds ratio was 1.99 (95% CI: 1.25–3.17, *p* = 0.004), indicating that patients with high psychological distress were approximately twice as likely to achieve successful weight loss. This association persisted at 12 months, with an adjusted odds ratio of 1.86 (95% CI: 1.02–3.39, *p* = 0.044), demonstrating that the relationship between preoperative psychological distress and weight loss success remained significant throughout the first postoperative year.

### 3.6. Subgroup Analysis by Sex

The association between psychological distress and successful weight loss was examined separately for male and female patients to address potential effect modification by sex (Table 3). Testing for interaction between sex and GHQ status did not reveal significant interaction effects at either time point (6 months: *p* for interaction = 0.553; 12 months: *p* for interaction = 0.583), suggesting that the association between psychological distress and weight loss outcomes did not significantly differ by sex.

In female patients, high psychological distress was significantly associated with successful weight loss at 6 months (*n* = 273; adj. OR 1.87, 95% CI: 1.07–3.28, *p* = 0.028), with the trend persisting at 12 months (*n* = 234; adj. OR 1.79, 95% CI: 0.89–3.62, *p* = 0.105). In male patients, the association showed a similar direction with comparable effect sizes, though not reaching statistical significance, likely reflecting the smaller sample size (6 months: *n* = 94, adj. OR 2.44, 95% CI: 0.96–6.21, *p* = 0.062; 12 months: *n* = 81, adj. OR 2.34, 95% CI: 0.67–8.26, *p* = 0.185). Notably, the point estimates for odds ratios were numerically similar or even higher in males compared to females at both time points, further supporting the lack of significant sex-based differences in the association. The lack of significant interaction, combined with consistent effect directions across both sexes, suggests that the relationship between preoperative psychological distress and successful weight loss is present in both male and female patients.

### 3.7. Post Hoc Power Analysis

Post hoc power analysis demonstrated that our study achieved 90.1% power to detect the observed association at 6 months and 58.2% power at 12 months.

### 3.8. Comparison of Retained vs. Lost to Follow-Up Patients

To assess potential attrition bias, we compared baseline characteristics between retained and lost patients at both follow-up time points (Table A2). At 6-month follow-up, 367 patients (79.1%) had available data, while 97 (20.9%) were lost to follow-up. Baseline characteristics were well-balanced between the two groups. Critically, GHQ scores—our primary exposure variable—were identical between groups (both median 7; retained: IQR 2–13; lost: IQR 4–15; *p* = 0.072). Similarly, baseline BMI (43.7 vs. 44.1 kg/m^2^, *p* = 0.487), preoperative weight (117.0 vs. 116.5 kg, *p* = 0.992), sex distribution (*p* = 0.273), surgery type (*p* = 0.149), and number of obesity-related comorbidities (*p* = 0.222) showed no significant differences. Only age showed a statistically significant but clinically minimal difference (median 34 vs. 33 years, 1-year difference, *p* = 0.045).

At 12-month follow-up, 315 patients (67.9%) had available data, while 149 (32.1%) were lost to follow-up. With greater attrition, several baseline differences emerged between retained and lost patients. Patients lost to follow-up were younger (median 32 vs. 35 years, *p* < 0.001), had different surgery type distributions (83.9% vs. 68.9% sleeve gastrectomy, *p* = 0.003), and fewer obesity-related comorbidities (*p* = 0.047). GHQ scores showed a borderline significant difference, with lost patients having numerically higher scores (median 8 vs. 6, *p* = 0.053). Baseline BMI (*p* = 0.161) and preoperative weight (*p* = 0.454) remained comparable between groups.

## 4. Discussion

Our study revealed an unexpected but significant relationship between preoperative psychological distress and weight loss outcomes following bariatric surgery. Contrary to our initial hypothesis that higher psychological distress would negatively affect post-surgical outcomes, we found that patients with elevated psychological distress (GHQ ≥ 6) were approximately twice as likely to achieve successful weight loss at both 6 and 12 months post-surgery (adj. OR 1.99 and 1.86, respectively) after adjusting for relevant confounders. This finding challenges the traditional view that psychological distress might negatively impact post-surgical outcomes and suggests a more complex relationship between mental health and weight loss success.

Several potential mechanisms might explain this counterintuitive finding. First, individuals with higher psychological distress may experience heightened body awareness and health-related anxiety, potentially leading to higher motivation to change or stricter adherence to post-surgical dietary and lifestyle recommendations. This hypothesis is supported by previous research showing that moderate levels of health anxiety can promote positive health behaviors and medical compliance [22,23]. Second, patients with elevated psychological distress in our program received additional psychological support and monitoring during the pre-surgical period, which may have equipped them with better coping strategies and emotional regulation skills for managing post-surgical challenges [14,24]. Third, the experience of psychological distress might reflect a greater emotional investment in the surgery’s success, translating into increased motivation for lifestyle changes and commitment to post-surgical care protocols [25,26,27].

The association between psychological distress and weight loss strengthened from 6 to 12 months post-surgery, possibly through sustained behavioral modifications and enhanced engagement with post-surgical care [26,27]. This finding is particularly noteworthy given that the 6–12 month period often represents a critical phase when initial rapid weight loss begins to plateau and patients must rely more heavily on behavioral strategies to maintain their progress [13,27,28]. However, weight loss may also be caused by the improvement of comorbidities after bariatric surgery [29].

Our findings contribute new insights to the complex relationship between psychological factors and bariatric surgery outcomes. Previous research has shown mixed results regarding this association. In a comprehensive systematic review, Dawes et al. (2016) found that the relationship between preoperative psychological distress and postoperative weight loss outcomes was inconsistent across studies, with some showing negative associations and others finding no significant relationship [12]. For instance, de Zwaan et al. (2011) conducted a prospective study using structured clinical interviews and found that preoperative anxiety and depression were common among bariatric surgery candidates [30]. However, their impact on surgical outcomes was not uniformly negative, suggesting that the relationship between psychological factors and weight loss success is more nuanced than previously thought. This aligns with our findings that higher psychological distress was associated with greater weight loss at both 6 and 12 months post-surgery.

The discrepancy between our results and earlier research might be explained by several factors. Our study used GHQ-28 as a comprehensive measure of psychological distress, capturing a broader range of psychological symptoms compared to instruments focused solely on depression or anxiety. Additionally, our Asian population might differ from Western cohorts in terms of psychological presentations and their relationship with weight management behaviors.

Interestingly, a recent longitudinal study (2025) in Taiwan [17] found that having a psychiatric disorder before surgery did not significantly affect a person’s weight change after the procedure. This is contrary to our findings and could be due to several factors. First, the study in Taiwan was conducted on Chinese patients in a developed country, which has different cultural and lifestyle factors from our participants. Thailand is a developing country, and our healthcare structure is not as advanced as Taiwan’s. Second, our study had a larger number of participants (*n* = 464) compared to the Taiwanese study (*n* = 147), which suggests our findings may be more robust in terms of statistical power. Third, the differing findings may stem from the specific psychiatric assessment tools used (GHQ-28 vs. Chinese Health Questionnaire/Taiwanese Depressive Questionnaire) and the different psychological constructs they measure. Given these conflicting results, a definitive conclusion cannot be drawn. Further large-scale studies are needed to explore the relationship between preoperative psychiatric disorders and post-surgical weight change.

The inconsistent findings across the literature may reflect several factors, including heterogeneity in psychological assessment tools (structured interviews vs. self-report questionnaires), timing of assessments, population differences (Western vs. Asian cohorts), and varying levels of psychological support provided within bariatric surgery programs. Our study’s use of a general psychological distress measure (GHQ-28) rather than diagnosis-specific tools may capture different dimensions of psychological functioning than studies focusing on specific psychiatric diagnoses. These methodological differences underscore the need for standardized approaches to psychological assessment in bariatric surgery research.

This study has several key strengths. It addresses an important clinical question about the relationship between preoperative psychological distress and bariatric surgery outcomes. The findings are particularly valuable as they challenge the traditional assumption that psychological distress is a barrier to successful outcomes. The study’s relatively large sample size of 464 patients provides adequate statistical power to detect meaningful associations while controlling for important confounders such as age, sex, surgery type, and baseline body weight. Furthermore, the study contributes valuable data from an Asian population, adding diversity to the predominantly Western-based bariatric surgery literature. This makes the findings particularly relevant for clinicians in the region. Finally, the study highlights the importance of integrating psychological care into standard bariatric surgery protocols, emphasizing that psychological assessment is not merely a screening tool but a valuable method for optimizing post-surgical outcomes through targeted support.

Several limitations of this study should be considered. Our single-center retrospective design may limit the generalizability of our findings to other settings or populations. The retrospective design of the study prevented the inclusion of potentially important socioeconomic factors, such as education, marital status, and employment status, in our analysis. It also limited our ability to obtain specific data on the personnel or equipment used for measurements, although the measurements were primarily conducted by nurses specializing in bariatric care.

Regarding statistical power and attrition, our study demonstrates important differential patterns across follow-up periods. At 6-month follow-up (79% retention), we achieved excellent statistical power (90.1%) and found no systematic differences in baseline characteristics between retained and lost patients, including GHQ scores (*p* = 0.072). This supports the validity of our 6-month findings as primary results. However, at 12-month follow-up (68% retention), achieved power was more modest (58.2%), and several baseline differences emerged: patients lost to follow-up were younger (*p* < 0.001), had different surgery type distributions (*p* = 0.003), fewer obesity-related comorbidities (*p* = 0.047), and borderline higher GHQ scores (*p* = 0.053).

This differential attrition pattern suggests potential selection bias in the 12-month analysis. If patients with higher psychological distress who were lost had achieved better outcomes (consistent with our hypothesis), our 12-month estimates may be conservative. Nevertheless, the 12-month association remained statistically significant despite this potential bias and modest power. The observed difference at 12 months (9.0 percentage points) was smaller than the minimum detectable effect with 80% power (12 percentage points), explaining the lower achieved power. Given these considerations, we emphasize the 6-month findings—where no systematic attrition bias was detected and statistical power was excellent—as our primary results, with 12-month findings providing supportive but cautiously interpreted secondary evidence.

The use of the GHQ-28, while a valuable screening tool for general psychological distress, captures only one aspect of a patient’s psychological state and should not replace a comprehensive psychological assessment. A broader range of psychological assessments would be needed to holistically observe patients in diverse dimensions such as anxiety, quality of life, or overall well-being [18,31]. Our exclusion of individuals with severe psychiatric disorders, a necessary step for patient safety, may also limit the generalizability of our findings to the full spectrum of mental health conditions seen in bariatric surgery candidates.

While our cohort was predominantly female (75.6%) and underwent primarily laparoscopic sleeve gastrectomy, these characteristics are common in bariatric surgery populations [29,32]. We addressed these factors by including sex and surgery type as covariates in our statistical models. Comprehensive sex-stratified analyses with formal interaction testing demonstrated consistent associations across both male and female patients, with no evidence of sex-based effect modification (*p* > 0.5 at both time points), supporting the generalizability of our findings across sexes.

The 12-month follow-up period may not fully capture long-term trajectories of weight loss and psychological adjustment. We lacked data on changes in psychological distress after surgery, preventing analysis of the reciprocal relationship between weight loss and mental health outcomes. Additionally, information on post-surgical compliance with dietary and lifestyle recommendations was not systematically collected, limiting our ability to explore the potential behavioral mechanisms behind our findings.

Future prospective studies with longer follow-up periods and predetermined sample sizes based on a priori power analyses are needed to validate these findings. These studies should also incorporate detailed assessments of mediating factors, such as eating behaviors, adherence, physical activity levels, and healthcare engagement patterns [16,33], and examine changes in psychological distress over time to understand the reciprocal relationship between mental health and weight loss outcomes.

Ongoing psychological assessment, not only before but also after bariatric surgery, is advised. This approach can help motivate lifestyle modifications, provide support for distress throughout treatment, and potentially prevent more severe psychiatric conditions. Our findings suggest that preoperative psychological distress should not be considered a barrier to surgery but rather a potential predictor of success, particularly when accompanied by appropriate support. Furthermore, long-term post-operative care, including adherence to dietary and lifestyle recommendations, encouraging behavioral interventions, or providing psychosocial support, is essential for achieving and sustaining weight loss [16,33].

## 5. Conclusions

In this large retrospective cohort study, we found that patients with high preoperative psychological distress achieved significantly greater weight loss following bariatric surgery compared to those with low distress, challenging traditional assumptions about psychological factors in surgical outcomes. Our findings suggest that preoperative psychological distress, rather than being a barrier to successful outcomes, may identify patients who are more likely to achieve significant weight loss when provided with appropriate support. These results support the importance of comprehensive pre-surgical psychological assessment, not as a barrier to surgery but as a valuable tool for optimizing post-surgical outcomes through targeted psychological support.

## Figures and Tables

**Table 1:** Patients’ characteristics.

	Total	Low Distress (GHQ < 6)	High Distress (GHQ ≥ 6)	*p*-Value
	*n* = 464	*n* = 194	*n* = 270	
Sex				***0.025***
Male	113 (24.4)	58 (29.9)	55 (20.4)	
Female	351 (75.6)	136 (70.1)	215 (79.6)	
Age (year), median (IQR)	34 (28, 42)	34 (27, 42)	33 (28, 40)	0.798
GHQ score, median (IQR)	7 (3, 13)	2 (1, 3.8)	12 (8, 17)	***<0.001***
Surgery types				0.534
Sleeve gastrectomy	342 (73.7)	148 (76.3)	194 (71.9)	
Roux-en-Y Gastric bypass	66 (14.2)	24 (12.4)	42 (15.6)	
SADI-S	56 (12.1)	22 (11.3)	34 (12.6)	
Obesity-related comorbidities				
Hypertension	153 (33)	68 (35.1)	85 (31.6)	0.497
Type 2 diabetes	135 (29.2)	62 (32)	73 (27.1)	0.307
Impaired fasting glucose	38 (8.2)	18 (9.3)	20 (7.4)	0.580
Dyslipidemia	169 (36.6)	74 (38.3)	95 (35.3)	0.570
Obstructive sleep apnea	99 (41.8)	37 (40.2)	62 (42.8)	0.801
Gastroesophageal reflux	52 (11.3)	19 (9.9)	33 (12.3)	0.519
MASLD	149 (32.1)	56 (28.9)	93 (34.4)	0.243
No. of obesity-related comorbidities				0.641
None	40 (8.6)	15 (7.7)	25 (9.3)	
1	71 (15.3)	32 (16.5)	39 (14.4)	
2	71 (15.3)	26 (13.4)	45 (16.7)	
3	89 (19.2)	33 (17)	56 (20.7)	
4	82 (17.7)	36 (18.6)	46 (17)	
5	55 (11.9)	28 (14.4)	27 (10)	
≥6	56 (12.1)	24 (12.4)	32 (11.9)	

Data are *n* (%), unless otherwise indicated. Bold *p*-values indicate statistically significant differences (*p* < 0.05).GHQ, General Health Questionnaire; SADI-S, Single anastomosis duodeno-ileal bypass; MASLD, metabolic associated steatotic liver diseases.

**Table 2:** Baseline body weight and post-operative weight loss outcomes.

	Total	Low Distress (GHQ < 6)	High Distress (GHQ ≥ 6)	*p* Value
	*n* = 464	*n* = 194	*n* = 270	
Pre-op BW (kg), median (IQR)	117 (103.2, 135.8)	118.9 (106.5, 140)	115 (101.9, 134.2)	0.056
Pre-op BMI (kg/m^2^), median (IQR)	43.9 (39.4, 50)	44.6 (39.7, 51)	43.3 (39, 49.1)	0.124
Post-op weight loss, median (IQR)				
6 months (kg) †	29.4 (24, 38.3)	29.5 (24.4, 38)	29.3 (23.9, 38.5)	0.912
12 months (kg) ‡	37 (29, 46.1)	37.2 (28.5, 44.8)	36.2 (29.4, 47)	0.627
%Total weight loss, mean (SD)				
6 months (%) †	25.8 (6.1)	25.1 (5.9)	26.4 (6.2)	0.054
12 months (%) ‡	31.6 (7.3)	30.7 (6.8)	32.3 (7.7)	0.055
Successful weight loss				
6 months †	193 (52.6)	70 (43.5)	123 (59.7)	***0.003***
12 months ‡	251 (79.7)	103 (74.6)	148 (83.6)	0.068

Data are *n* (%), unless otherwise indicated. Bold *p*-values indicate statistically significant differences (*p* < 0.05). † Patients completed 6 months follow-up (*n* = 367), with low distress (*n* = 161) and high distress (*n* = 206). ‡ Patients completed 12 months follow-up (*n* = 315), with low distress (*n* = 138) and high distress (*n* = 177). GHQ, General Health Questionnaire; BW, body weight; BMI, body mass index.

**Table 3:** Subgroup analysis by sex for successful weight loss.

	6 Months		12 Months	
	Adj. OR (95% CI)	*p*-Value	Adj. OR (95% CI)	*p*-Value
Female	*n* = 273		*n* = 234	
High distress	1.87 (1.07, 3.28)	***0.028***	1.79 (0.89, 3.62)	0.105
Male	*n* = 94		*n* = 81	
High distress	2.44 (0.96, 6.21)	0.062	2.34 (0.67, 8.26)	0.185
*p* for interaction		0.553		0.583

Adjusted for age, surgery type, number of obesity-related comorbidities, and baseline body weight. Bold *p*-values indicate statistically significant results (*p* < 0.05). *p*-value for interaction between sex and GHQ status is 0.553 and 0.583 for SWL at 6 months and 12 months respectively. Adj. OR, adjusted odds ratio; CI, confidence interval.

## Data Availability

The datasets generated during and/or analyzed in this study are not publicly available due to privacy but can be obtained upon request from the corresponding author.

## Abbreviation

The following abbreviations are used in this manuscript:

BMI	Body mass index
BW	Body weight
GHQ	General Health Questionnaire
SADI-S	Single anastomosis duodeno-ileal bypass
MASLD	Metabolic Associated Steatotic Liver Diseases
OR	Odds Ratio
SWL	Successful weight loss
TWL	Total weight loss

## Appendix A

**Table A1:** Multivariable logistic regressions for achieving successful weight loss.

	6 Months (*n* = 367)		12 Months (*n* = 315)	
	Adj. OR (95% CI)	*p*-Value	Adj. OR (95% CI)	*p*-Value
High distress	1.99 (1.25, 3.17)	0.004	1.86 (1.02, 3.39)	0.044

Adjusted odds ratio (95% CI) of GHQ status (high distress [GHQ ≥ 6] vs. low [GHQ < 6]). Regression models were adjusted for age, sex, surgery type, number of obesity-related comorbidities, and baseline body weight. GHQ, General Health Questionnaire; Adj. OR., adjusted odds ratio.

**Table A2:** Comparison of baseline characteristics between patients retained and lost to follow-up.

Characteristic	6-Month Follow-Up			12-Month Follow-Up		
	Retained (*n* = 367)	Lost (*n* = 97)	*p*-Value	Retained (*n* = 315)	Lost (*n* = 149)	*p*-Value
Age (years), median (IQR)	34 (28, 42)	33 (26, 36)	0.045	35 (29, 43)	32 (26, 36)	<0.001
Sex			0.273			0.38
Male	94 (25.6)	19 (19.6)		81 (25.7)	32 (21.5)	
Female	273 (74.4)	78 (80.4)		234 (74.3)	117 (78.5)	
BMI (kg/m^2^), median (IQR)	43.7 (39.1, 50.0)	44.1 (40.6, 49.6)	0.487	43.5 (39.0, 49.5)	44.2 (40.6, 51.2)	0.161
Weight (kg), median (IQR)	117.0 (103.3, 136.2)	116.5 (103.0, 132.0)	0.992	117.9 (102.2, 135.5)	117.0 (104.0, 137.0)	0.454
GHQ score, median (IQR)	7 (2, 13)	7 (4, 15)	0.072	6 (2, 12)	8 (3, 14)	0.053
Surgery type			0.149			0.003
Sleeve gastrectomy	263 (71.7)	79 (81.4)		217 (68.9)	125 (83.9)	
Roux-en-Y Gastric bypass	56 (15.3)	10 (10.3)		53 (16.8)	13 (8.7)	
SADI-S	48 (13.1)	8 (8.2)		45 (14.3)	11 (7.4)	
No. of comorbidities			0.222			0.047
None	27 (7.4)	13 (13.4)		22 (7.0)	18 (12.1)	
1	55 (15.0)	16 (16.5)		42 (13.3)	29 (19.5)	
2	57 (15.5)	14 (14.4)		52 (16.5)	19 (12.8)	
3	70 (19.1)	19 (19.6)		64 (20.3)	25 (16.8)	
4	70 (19.1)	12 (12.4)		57 (18.1)	25 (16.8)	
5	40 (10.9)	15 (15.5)		33 (10.5)	22 (14.8)	
≥6	48 (13.1)	8 (8.2)		45 (14.3)	11 (7.4)	

Data are *n* (%), unless otherwise indicated. GHQ, General Health Questionnaire; BMI, body mass index; IQR, interquartile range.
